# Neuropsychiatric symptoms and arachnoid cysts: tracing the association regarding a case report

**DOI:** 10.1192/j.eurpsy.2024.889

**Published:** 2024-08-27

**Authors:** C. Regueiro Martín-Albo, F. M. Sanabria, E. E. Durán, M. F. Fariña, N. R. Criado

**Affiliations:** Hospital Clínico San Carlos, Madrid, Spain

## Abstract

**Introduction:**

In the review of recent literature, we found very few presentations of case reports in which the presumed association between psychiatric disorders and arachnoid cysts is discussed. Arachnoid cysts are rare brain tumors with little apparent symptomatic impact and in most cases, they are diagnosed incidentally. We present the case of a 15-year-old adolescent with a personal history of a previous severe depressive episode, as well as suspicion of serious mental pathology in the family. It presents a subacute onset episode, in the context of regular cannabis consumption, consisting of intense emotional lability and psychomotor restlessness, a tendency toward irritability, decreased sleep needs, and the appearance of delusional ideas of harm and self-referential interruption. During the study of the case, and incidentally, the cranial magnetic resonance imaging revealed the presence of an arachnoid cyst located in the left frontotemporal location, approximately 4 cm in diameter.

**Objectives:**

(1) To describe the clinical particularities of this case, focusing on the diagnostic difficulties we faced. (2) To review current scientific evidence regarding the possible association between neuropsychiatric symptoms and arachnoid cysts.

**Methods:**

A review of the patient’s clinical history was carried out and complementary tests were performed. Likewise, a review of the available scientific literature was also performed in relation to the appearance of neuropsychiatric symptoms associated with the presence of arachnoid cysts.

**Results:**

The literature regarding the possible association between psychosis and arachnoid cysts is scarce. However, it is proposed that arachnoid cysts may be associated with various neuropsychiatric alterations, such as affective alterations, schizophrenia-like psychosis or amnestic symptoms. The atypicality in the symptoms sometimes leads us to suspect an organic origin of the condition, with some features such as associated memory deficits, emotional incontinence, movement disorders or neurological focal data; some of which are present in the case at hand.

**Conclusions:**

There is controversy among different sources regarding the role of the cyst in the development of symptoms or, on the contrary, its presentation only as a chance finding. Further investigation focusing on clinical observations and neuroimaging is needed.

**Disclosure of Interest:**

None Declared

